# Genomic Analysis of Inbreeding Depression on Productive Traits in Rubia Galega Beef Cattle Breed

**DOI:** 10.1111/jbg.70034

**Published:** 2025-12-16

**Authors:** N. Mejuto‐Vázquez, C. Hervás‐Rivero, R. Rodríguez‐Bermúdez, D. López‐Carbonell, M. Hermida, P. Martínez, L. Varona

**Affiliations:** ^1^ Departamento de Anatomía, Produción Animal e Ciencias Clínicas Veterinarias Facultade de Veterinaria, Universidade de Santiago de Compostela Lugo Spain; ^2^ Departamento de Zooloxía, Xenética e Antropoloxía Física Facultade de Veterinaria, Universidade de Santiago de Compostela Lugo Spain; ^3^ Universidad de Zaragoza, Instituto Agroalimentario de Aragón (IA2) Zaragoza Spain

**Keywords:** BLUP, indigenous, mstn, run of homozygosity, SNP

## Abstract

In autochthonous livestock breeds with small populations, such as the Rubia Galega from Galicia (Spain), mating between relatives is common and can lead to inbreeding depression. Genomic inbreeding coefficients were estimated for 4984 animals using ~63,000 SNPs to assess inbreeding depression in four key traits: age at first calving (AFC) with 3503 records, calving interval (CI) with 3315 records, birth weight (BW) with 4878 records and weight at 210 days (W210) with 3285 records. Runs of homozygosity were sorted by length ([1,2], (2,4], (4,8], (8,16], > 16 Mb), and the corresponding inbreeding coefficients (*F*_ROH>1_, *F*_ROH>2_, *F*_ROH>4_, *F*_ROH>8_, *F*_ROH>16_) were calculated using the *consecutiveRUNs* R package. A Genomic BLUP (GBLUP) was conducted for each *F*_ROH_ estimate using the *BLUPF90+* programs. The results revealed significant inbreeding depression for AFC and CI, whereas W210 and BW exhibited similar inbreeding trends, but the effects of inbreeding on these traits were not statistically significant. To further explore the genetic basis of inbreeding depression, SNPs located within ROHs were tested, though a *t*‐test, for their association with phenotypic traits. Genes located in significant regions (−log(*p*‐value) > 3 from *t*‐test) were annotated using Ensembl BioMart within a ± 0.5 Mb window. Recent inbreeding (ROH > 8 Mb) showed significant negative effects on reproductive traits, and key genomic regions—particularly on chromosome 2 involving *MSTN*, *NAB1*, and *COL5A2*—were linked to increased AFC and reduced BW and W210; ROH‐based inbreeding estimates proved effective in detecting inbreeding depression in this native breed. Overall, ROH‐based analyses revealed genomic regions and candidate genes, notably *MSTN*, contributing to inbreeding depression and key production traits in Rubia Galega cattle.

## 1 Introduction

Rubia Galega (RG) cattle represent one of the largest autochthonous beef cattle populations on the Iberian Peninsula, especially in the Galicia region (Northwest Spain). Since the 1990s, the breeding programme has focussed on improving growth rate and body conformation while preserving calving ease and meat quality. However, due to limited population size and suboptimal management practices, mating between relatives can sometimes occur. This can result in reduced fitness and a decline in the performance of key traits, a phenomenon known as inbreeding depression, which affects all livestock species (Leroy 2014).

Artificial selection and the widespread use of reproductive technologies have intensified the problem of inbreeding in cattle worldwide, negatively impacting both reproductive and productive performance (Cortes et al. 2024; Forneris et al. 2021; Nishio et al. 2023). According to Doekes et al. (2021), each 1% increase in the inbreeding coefficient (*F*) is associated with a 0.13% decrease in average trait performance. This decline is attributed to a rise in genome‐wide homozygosity, which triggers two processes that diminish animal performance: (i) an increased frequency of homozygous recessive alleles, and (ii) higher homozygosity at loci with heterozygote advantage (overdominance) (Charlesworth and Willis 2009).

Several methods exist for estimating the inbreeding coefficient, defined as the probability that a pair of alleles is identical by descent (IBD) (Bjelland et al. 2013). These methods can be used for designing mating strategies or assessing inbreeding depression in livestock populations. One widely used metric is the pedigree‐based inbreeding coefficient (***F***_PED_), which relies heavily on the quality of pedigree records (Dadousis et al. 2022; Sørensen et al. 2008). However, this method may underestimate inbreeding in populations undergoing artificial selection (Nietlisbach et al. 2017), as it does not account for the higher relatedness among selected animals compared to the average population (Caballero et al. 2022).

The development of new genomic technologies has enabled the identification of millions of genetic markers, such as SNPs, facilitating the creation of potentially more accurate inbreeding coefficients that account for Mendelian sampling segregation (Bjelland et al. 2013; Doekes et al. 2019; Hill and Weir 2011). Two such coefficients are *F*_GMD_ and *F*_ROH_. *F*_GMD_ is calculated using the diagonal of the genomic relationship matrix (**G**) (VanRaden 2008) minus one, whereas *F*_ROH_ is based on the identification of runs of homozygosity (ROH).

ROH are autozygous segments inherited from a common ancestor (Ceballos et al. 2018), and the ROH‐derived inbreeding coefficient (***F***_ROH_) is defined as the total length of the genome within ROHs divided by the total length of genome covered with SNPs (McQuillan et al. 2008). This method effectively identifies homozygosity and may help to approximate the distinction between IBD and identity by state (IBS), which a simple measure of the percentage of homozygous alleles cannot accomplish (Bjelland et al. 2013). Additionally, *F*_ROH_ enables the distinction between ancient and recent inbreeding based on the length of ROHs. More recent ROHs tend to be longer and are generally associated with more severe negative effects, whereas older ROHs are shorter and may have lesser impact on inbreeding depression due to purging (García‐Dorado 2012). However, both types may contribute to inbreeding depression (Doekes et al. 2019).

In this study, we used runs of homozygosity (ROHs) in Rubia Galega cattle to estimate inbreeding, assess the impact of inbreeding depression on four economically important traits and identify candidate genes associated with inbreeding depression or genetic variability in these traits.

## 2 Material and Methods

### 2.1 Genomic Data and Quality Control

Genomic data were obtained from 4984 RG animals collected in Galicia (NW Spain), belonging to Asociación Nacional de Criadores de Ganado Vacuno Selecto de Raza Rubia Gallega (ACRUGA). All individuals were genotyped with the Axiom Bovine Genotyping v3 Array (384HT format), which includes ~63,000 SNPs. Markers not mapped to autosomes were excluded. SNPs with a call rate below 90% were removed, as well as animals with less than 80% of the SNPs genotyped. This threshold was selected to ensure a balance between genotyping quality and maintaining an adequate sample size, as stricter filters would have excluded a considerable number of individuals (e.g., a 90% threshold would have removed 186 animals). Filtering by minor allele frequency (MAF) was not performed because, although rare alleles are typically found in the heterozygous state under Hardy–Weinberg equilibrium (HWE), in homozygous regions resulting from inbreeding, they are often observed in homozygosity. Removing them could disrupt homozygosity blocks. Additionally, fixed or nearly fixed regions often exhibit low allele frequencies. Furthermore, a HWE filter was not applied due to the agricultural population under the influence of selection dynamics, assisted reproduction, etc. (Ceballos et al. 2018).

We also used the pedigree of the RG population from ACRUGA that comprises 522,885 animals, of which, 61,890 (11.84%) lack information about any ancestor and 141,878 (27.13%) lack information about one parent. The *optiSel* (version 2.0.9.) R (R version 4.3.2 [2023‐10‐31 ucrt]) package (Wellmann and Pfeiffer 2009) was used to calculate the pedigree completeness index (PCI) (average = 0.50) (MacCluer et al. 1983), the number of equivalent complete generations (ECG) (average = 3.79) and the full complete generations (FCG) (average = 1.64). The pedigree has been corrected, as the ACRUGA association utilises paternity testing. The main issue, however, is the lack of information—primarily regarding the grandparents and great‐grandparents of many individuals. The molecular genetic relatedness is quite high (0.76), but the absence of more distant ancestral relationships complicates the calculations.

### 2.2 ROH Calculation

ROHs were identified using the consecutive method (Marras et al. 2015) from the R package detectRUNS, which detects stretches of consecutive SNPs in homozygosity across entire chromosomes. This method identifies ROHs as uninterrupted sequences of homozygous SNPs, allowing for a small number of heterozygous or missing calls if specified. It was chosen over the sliding windows used by PLINK (Purcell et al. 2007), which defines a window of a fixed number of SNPs and calculates the proportion of homozygous markers within it as the window shifts along the genome, because of its limitations: Sliding windows can introduce artificial runs and may fail to detect segments longer than the window size (Ferenčaković et al. 2013).

A ROH was defined as a segment containing 15 or more SNPs, with no gaps longer than 1 Mb between them, to prevent the inclusion of heterozygous hidden SNPs (Powell et al. 2010). Additionally, only ROHs longer than 1 Mb were considered, following the criteria recommended by Purfield et al. (2012).

The allowed number of missing SNPs and heterozygous SNPs varied among the ROH length classes (in Mb), which were categorised into five groups: [1, 2], (2, 4], (4, 8], (8, 16], and > 16, following the recommendations of Ferenčaković et al. (2013b). Here, square brackets [] indicate that the boundary value is included in the interval, whereas parentheses () indicate that it is excluded. The initial step involved conducting the analysis with fixed parameters, without missing or heterozygous SNPs. In a second step, the limits were then established based on the average results for each class (Table 1).

**TABLE 1:** Number of heterozygous (het) and missing single nucleotide polymorphisms allowed within runs of homozygosity (ROHs).

	ROH's categories by length (in Mb)				
	[1,2]	(2,4]	(4,8]	(8,16]	> 16
Mean (in SNPs)	26.91	60.06	120.86	226.65	431.43
Het allowed	0	0	0	1	1
Missing allowed	0	1	1	2	4

Abbreviations: ROH, run of homozygosity; SNP, single nucleotide polymorphism.

The allowed number of heterozygous SNPs was calculated based on the assumed genotype error rate of 0.25% (Ferenčaković et al. 2013). This led to the following restrictions for each class: no heterozygous SNPs for [1, 2], (2, 4] and (4, 8]; one heterozygous SNP for (8, 16] and for > 16 (Table 1). To determine the acceptable number of missing SNPs, the overall missing rate across the genome was calculated, yielding a rate of 1.04%. Based on this, the restrictions for each class were established as follows: no missing SNPs for [1, 2]; one missing SNP for (2, 4] and (4, 8]; two missing SNPs for (8, 16]; and four missing SNPs for > 16.

### 2.3 Inbreeding Coefficients

Three inbreeding coefficients were estimated for the genotyped individuals: (i) the pedigree‐based (*F*_PED_) calculated using the INBUPGF90 software (Aguilar and Misztal 2008) with the ACRUGA pedigree database; (ii) the genomic relationship‐based (*F*_GRM_), calculated with the *BLUPF90+* software (Misztal et al. 2018), and (iii) the *F*_ROH_ coefficient, based on the extent of genome coverage by ROH, assessed both overall (*F*_ROH_) and over specific length classes (*F*_ROH>2_, *F*_ROH>4_, *F*_ROH>8_ and *F*_ROH>16_). Pearson and Spearman correlations between the different inbreeding coefficients were calculated.

### 2.4 Inbreeding Depression

The impact of inbreeding was assessed on four key productive traits: Calving Interval (CI), Age at First Calving (AFC), Birth Weight (BW) and Weight at 210 days (W210). Data for BW were recorded for 4878 genotyped individuals, whereas AFC, CI and W210 data were available for 3503, 3315 and 3285 genotyped animals, respectively. The mean and the standard deviation of these data are presented in Table 2.

**TABLE 2:** Mean and standard deviation of AFC, CI (in days), BW and W210 (in kg).

Statistic	AFC	CI	BW	W210
Mean	850.85	390.54	42.65	300.83
SD	143.86	55.33	6.39	46.89
Min	552	321	30	158
Max	1569	599	72	465

Abbreviations: AFC, age at first calving; BW, birth weight; CI, calving interval; W210, weight at 210 days.

Inbreeding depression was estimated using the Genomic Best Linear Unbiased Prediction (GBLUP) method (VanRaden 2008), implemented via the BLUPF90+ software (Misztal et al. 2018), where the required variance components were estimated using the Average Information Restricted Maximum Likelihood (AIREML) algorithm (Gilmour et al. 1995). The procedure was repeated for each inbreeding coefficient based on runs of homozygosity (*F*_ROH_, *F*_ROH>2_, *F*_ROH>4_, *F*_ROH>8_ and *F*_ROH>16_) and was applied separately to each trait (BW, AFC, W210 and CI). The statistical models used for the analyses were:

$$y_{\mathrm{BW}}=c_{\mathrm{BW}}F+Xb_{\mathrm{BW}}+Wh_{\mathrm{BW}}+Zu_{\mathrm{BW}}+e_{\mathrm{BW}}$$

$$y_{\mathrm{AFC}}=c_{\mathrm{AFC}}F+Xb_{\mathrm{AFC}}+Wh_{\mathrm{AFC}}+Zu_{\mathrm{ACF}}+e_{\mathrm{ACF}}$$

$$y_{W210}=c_{W210}F+Xb_{W210}+Wh_{210}+Zu_{W210}+e_{W210}$$

$$y_{\mathrm{CI}}=c_{\mathrm{CI}}F+Xb_{\mathrm{CI}}+Wh_{\mathrm{CI}}+Zu_{\mathrm{CI}}+Wp_{\mathrm{CI}}+e_{\mathrm{CI}}$$

where $y_{\mathrm{BW}},y_{\mathrm{AFC}},y_{W210}$ and $y_{\mathrm{CI}}$ were the vectors of phenotypic records; $c_{\mathrm{BW}}$,$c_{\mathrm{AFC}},c_{W210}$ and $c_{\mathrm{CI}}$ were covariates with the vector ***F*** = {*F*_ROH_, *F*_ROH>2_, *F*_ROH>4_, *F*_ROH>8_ and *F*_ROH>16_} that contained the individual estimates of inbreeding; $b_{\mathrm{BW}},b_{\mathrm{AFC}},b_{W210}$ and $b_{\mathrm{CI}}$ were the vectors of systematic effects that included age as covariate for **W210**, sex for **BW** and **W210**, order of parity for CI and season‐year for all four traits; $h_{\mathrm{BW}},h_{\mathrm{AFC}},h_{W210}$ and $h_{\mathrm{CI}}$ were the random herd effects; $u_{\mathrm{BW}},u_{\mathrm{AFC}},u_{W210}$ and $u_{\mathrm{CI}}$ were the random additive genetic effects, $p_{\mathrm{CI}}$ was a random permanent environmental effect associated to each individual, as cows may have several records along its productive life. Finally, $e_{\mathrm{BW}},e_{\mathrm{AFC}},e_{W210}$ and $e_{\mathrm{CI}}$ were the vector of residuals. For **X** = {**BW**, **AFC**, and **W210**} the (co) variances between the random effects were:

$$\mathrm{Var}\left(\begin{array}{c} h_{X}\\ u_{X}\\ e_{X} \end{array}\right)=\left(\begin{array}{ccc} I\sigma_{\mathrm{hX}}^{2} & 0 & 0\\ 0 & G\sigma_{\mathrm{uX}}^{2} & 0\\ 0 & 0 & I\sigma_{\mathrm{eX}}^{2} \end{array}\right)$$

Moreover, for **CI** the (co) variances were:

$$\mathrm{Var}\left(\begin{array}{c} h_{\mathrm{CI}}\\ u_{\mathrm{CI}}\\ p_{\mathrm{CI}}\\ e_{\mathrm{CI}} \end{array}\right)=\left(\begin{array}{cccc} I\sigma_{\mathrm{hCI}}^{2} & 0 & 0 & 0\\ 0 & G\sigma_{\mathrm{uCI}}^{2} & 0 & 0\\ 0 & 0 & I\sigma_{\mathrm{pCI}}^{2} & 0\\ 0 & 0 & 0 & I\sigma_{\mathrm{eCI}}^{2} \end{array}\right)$$

where **I** is the identity matrix and **G** is the genomic relationship matrix (VanRaden 2008). Moreover, $\sigma_{\mathrm{hX}}^{2},\sigma_{\mathrm{uX}}^{2}$, $\sigma_{\mathrm{pX}}^{2}$ and $\sigma_{\mathrm{eX}}^{2}$ are the herd, additive genetic, permanent environmental and residual variances for **X** = {**BW**, **AFC**, **W210 and CI**).

### 2.5 Genomic Architecture of Inbreeding Depression

Finally, we explored the genetic architecture of inbreeding depression. To do this, we used the estimated residuals ($e_{\mathrm{BW}},e_{\mathrm{AFC}},e_{W210}$ and $e_{\mathrm{CI}}$) from the GBLUP model incorporating *F*_ROH_. The SNPs for each individual were coded as 1 if they were located within a ROH and as 0 if they were not. A *t*‐test was performed for each SNP to test the null hypothesis that the residuals from the GBLUP model do not differ between individuals with the SNP located within a ROH and those out of it. The SNPs showing the strongest statistical evidence (lowest *p* values) in the *t*‐test were considered the most significant results and were validated using an additional GBLUP model, which included the effects of the presence or absence of the specific SNP, along with the *F*_ROH_ calculated using all SNP markers except that one (Hervás‐Rivero et al. 2023).

As this was not a primary objective of the study, gene mining was conducted in a preliminary and exploratory manner, aiming to provide potential insights for future, more targeted research. BioMart from Ensembl (Martin et al. 2023) was subsequently used to retrieve annotated genes from the cattle genome, applying a ± 0.5 Mb window around the SNPs with a −log_10_(*p*‐value from *t*‐test) > 3. For a preliminary search of the biological function of these genes and their potential relationship to the observed traits, functional annotation and enrichment analysis were performed using DAVID (Database for Annotation, Visualisation, and Integrated Discovery) (Huang et al. 2009; Sherman et al. 2022) and g:GOSt function of g:Profiler software (Kolberg et al. 2023). As the functional annotation databases are based on the current *Bos taurus* reference genome (ARS‐UCD2.0), and the Axiom Bovine Genotyping v3 Array used in this study is referred to a previous version, a BLAST (Basic Local Alignment Search Tool) was performed to map the significant genomic regions identified in our analysis onto the corresponding regions in the current reference genome for each chromosome. This step ensured compatibility with current annotation tools and improved the biological interpretation of our results.

## 3 Results

### 3.1 ROH Calculations

A total of 393,877 ROHs, each containing 15 or more SNPs, were identified in the RG breed. The shortest ROH was 1 Mb with 25 SNPs, whereas the longest extended across 62.464 Mb with 1226 SNPs. On average, ROHs measured 2.531 Mb and included 53.87 SNPs. ROHs accounted for 8.4% of the cattle genome and were classified by length as follows: 67.30% were between 1 and 2 Mb, 18.00% between 2 and 4 Mb, 8.73% between 4 and 8 Mb, 4.44% between 8 and 16 Mb and 1.53% exceeding 16 Mb (Table 3).

**TABLE 3:** Number of runs of homozygosity (ROHs) by length category and their mean length in Mb and SNPs, along with their contribution to total ROH length by size class.

Class	*n* ROHs	Mean (Mb)	Mean (SNPs)	% total
[1,2]	265,093	1,301,742	26.31	67.30%
(2,4]	70,888	2,766,255	60.17	18.00%
(4,8]	34,389	5,475,561	122.41	8.73%
(8,16]	17,476	10,763,973	237.08	4.44%
> 16	6031	22,114,370	485.96	1.53%
Total	393,877	2,688,242	57.18	100.00%

Abbreviation: ROH, runs of homozygosity.

### 3.2 Inbreeding Coefficients

The analysis of *F*_ROH_, revealed that most inbreeding in the population stems from older events, represented by small homozygosity segments (> 1 Mb), with a mean *F* = 0.084 ± 0.049 (Table 4). As the size of ROHs increases, reflecting more recent inbreeding, the average *F* decreases. Notably, some animals displayed no evidence of recent inbreeding (*F* = 0).

**TABLE 4:** Mean, maximum, and minimum value of the inbreeding coefficient based on runs of homozygosity categories.

Statistic	Runs of homozygosity category by length (in Mb)				
	> 1 (total)	> 2	> 4	> 8	> 16
Mean	0.084	0.057	0.041	0.026	0.011
SD	0.049	0.046	0.041	0.032	0.019
Max	0.489	0.442	0.404	0.363	0.255
Min	0.001	0	0	0	0

**FIGURE 1:**
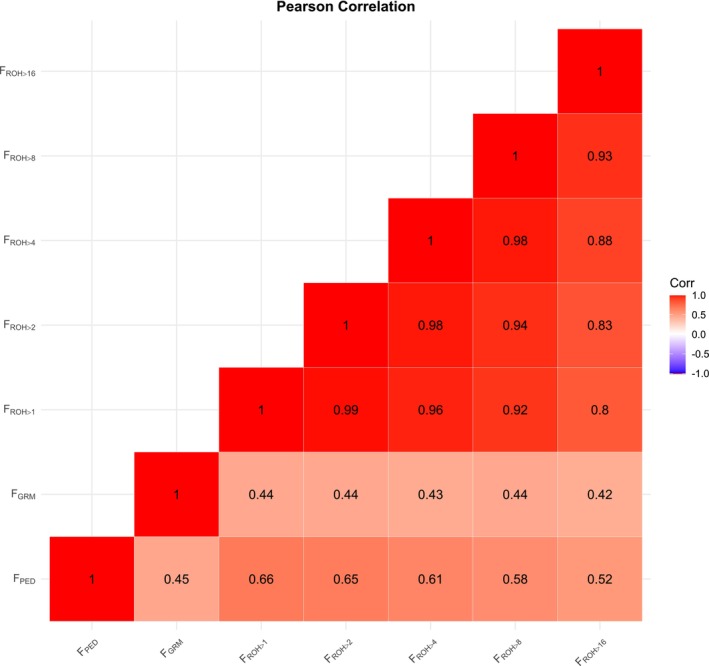
Pearson correlations between inbreeding coefficients (*F*_ROH_, *F*_PED_, *F*_GRM_) and *F*_ROH_ calculated using different ROH length thresholds. *F*_PED_: Inbreeding coefficient based on pedigree; *F*_GRM_: Inbreeding coefficient based on genomic relationship matrix; *F*_ROH>1_: Inbreeding coefficient based on ROHs great than 1 Mb; *F*_ROH>2_: Inbreeding coefficient based on ROHs great than 2 Mb; *F*_ROH>4_: Inbreeding coefficient based on ROHs great than 4 Mb; *F*_ROH>8_: Inbreeding coefficient based on ROHs great than 8 Mb; *F*_ROH>16_: Inbreeding coefficient based on ROHs great than 16 Mb. [Colour figure can be viewed at wileyonlinelibrary.com]

The strongest correlations (Pearson and Spearman) between inbreeding coefficients were observed between *F*_ROH_ and *F*_ROH>2_ (Spearman's *ρ* = 0.99, Pearson's *r* = 0.99) (Figure 1). Correlations gradually decreased as the ROH size increased, reaching a minimum of 0.71 (Spearman) and 0.80 (Pearson) for ROHs longer than 16 Mb. A similar trend was seen for correlations between inbreeding coefficients based on different ROH size categories, with the highest correlations found for smaller ROHs. The Spearman and Pearson correlations between *F*_ROH_ and *F*_GRM_, *F*_ROH_ and *F*_PED_, and *F*_GRM_ and *F*_PED_ were, respectively, 0.12 and 0.44, 0.52 and 0.66, and 0.00 and 0.44.

### 3.3 Inbreeding Depression

The results of the variance component estimation are presented in the Table 5. The covariate estimates for inbreeding derived from GBLUP indicate a linear increase in the undesirable impact of inbreeding as ROH size increased, except for BW, where the > 4 Mb ROH category showed a better estimate than the > 1 Mb and > 2 Mb categories (Table 6). For the other traits, the impact of inbreeding is evident, with a variation between ancient (> 1 Mb) and recent (> 16 Mb) inbreeding of: 114.37 days in AFC, 50.66 days in CI and 70.67 kg in W210.

**TABLE 5:** Variance component estimates and heritability for reproductive and growth traits obtained from GBLUP analysis.

Effects	AFC	CI	BW	W210
*σ* ^2^_*hX*_	4659.2	130.3	7.704	216.73
*σ* ^2*p*^_*X*_		179.06		
*σ* ^2*u*^_*X*_	1934.3	114.93	3.354	156.03
*σ* ^2*e*^_*X*_	13221.0	2536.5	25.54	1128.5
*h* ^2^	0.31	0.046	0.15	0.17

Abbreviations: *σ* ^*2*^_*hX*_, random herd effect; *σ* ^*2p*^_*X*_, random permanent environmental effect; *σ* ^*2u*^_*X*_, random additive genetic effect; *σ* ^*2e*^_*X*_, residual effect; AFC, age at first calving; BW, birth weight; CI, calving interval; *h* ^*2*^, heritability; W210, weight at 210 days.

**TABLE 6:** Estimated effects of ROH‐based inbreeding on reproductive and weight traits.

Trait	Statistic	Class by length (in Mb)				
		> 1	> 2	> 4	> 8	> 16
AFC	Estimate	130.48	141.37	156.77	201.07	244.85
	*p*	0.0068	0.0058	0.0055	0.0049	0.0227
CI	Estimate	28.89	30.14	31.91	45.89	79.55
	*p*	0.0130	0.0141	0.0168	0.0076	0.0032
BW	Estimate	0.64	−0.49	1.22	−2.41	−8.35
	*p*	0.55	0.46	0.62	0.3	0.083
W210	Estimate	9.61	−3.22	−15.79	−31.83	−61.06
	*p*	0.59	0.47	0.33	0.22	0.13

Abbreviations: AFC, age at first calving; BW, birth weight; CI, calving interval; ROH, runs of homozygosity; W210, weight at 210 days.

The statistical significance of these effects was assessed using *t*‐tests comparing residuals of individuals carrying specific SNPs within ROHs to those without, with further validation via a complementary GBLUP model including SNP presence/absence effects alongside *F*_ROH_ estimates excluding the tested SNP. For reproductive traits (CI and AFC), the undesirable increase in days was statistically significant (*p* < 0.05) when considering recent inbreeding; in particular, the *p* value was below 0.005 for *F*_ROH_>8 in AFC and *F*_ROH_>16 in CI. For growth traits, measured in kg, the results were not statistically significant, although a similar negative trend of inbreeding effects was observed for W210 and BW with ROH sizes greater than 8 Mb.

**FIGURE 2:**
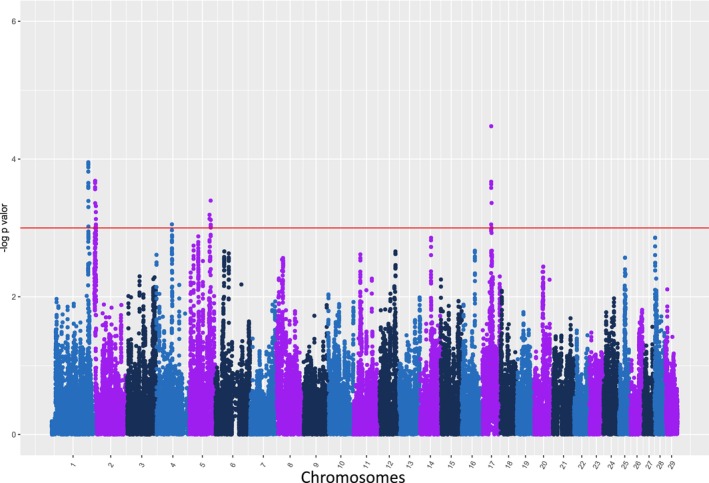
Significance of the presence or absence of SNPs within ROH for age at first calving. SNPs across the 29 autosomal chromosomes on the *x*‐axis, and significance as −log_10_(*p*‐value) on the *y*‐axis. Red line indicates the threshold at value 3 (*p* < 0.001). [Colour figure can be viewed at wileyonlinelibrary.com]

**FIGURE 3:**
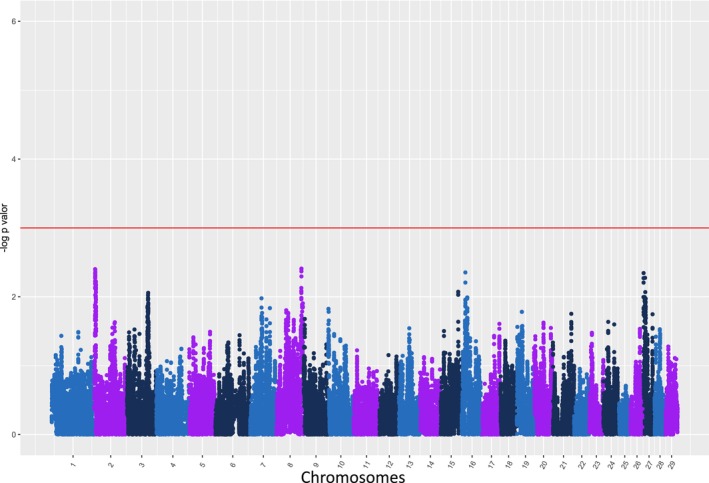
Significance of the presence or absence of SNPs within ROH for calving interval. SNPs across the 29 autosomal chromosomes on the *x*‐axis, and significance as −log_10_(*p*‐value) on the *y*‐axis. Red line indicates the threshold at value 3 (*p* < 0.001). [Colour figure can be viewed at wileyonlinelibrary.com]

Significant associations between AFC and five genomic regions were suggested by the absence of SNPs within a ROH (Figure 2). Three regions, located on Chromosomes 1, 5, and 17, were associated with a negative impact on the trait, leading to an increase in the age at first calving. Conversely, two regions on Chromosomes 2 and 4 showed a positive effect, reducing the age at first calving. No significant associations were observed for the CI trait (Figure 3).

**FIGURE 4:**
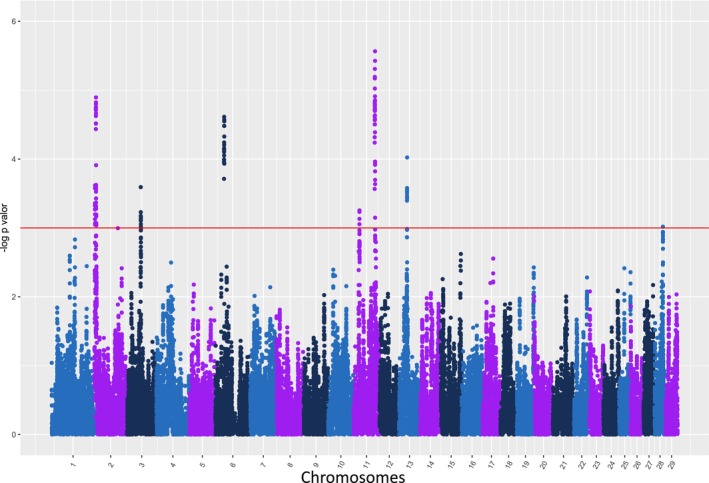
Significance of the presence or absence of SNPs within ROH for birth weight. SNPs across the 29 autosomal chromosomes on the *x*‐axis, and significance as −log_10_(*p*‐value) on the *y*‐axis. Red line indicates the threshold at value 3 (*p* < 0.001). [Colour figure can be viewed at wileyonlinelibrary.com]

**FIGURE 5:**
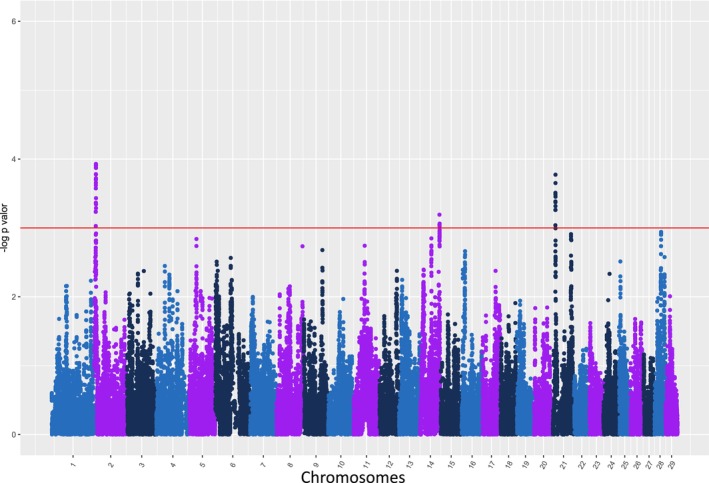
Significance of the presence or absence of SNPs within ROH for weight at 210 days. SNPs across the 29 autosomal chromosomes on the *x*‐axis, and significance as −log_10_(*p*‐value) on the *y*‐axis. Red line indicates the threshold at value 3 (*p* < 0.001). [Colour figure can be viewed at wileyonlinelibrary.com]

Significant associations were detected in three regions for W210; one on Chromosome 2 showed a negative effect (decreased weight), whereas the other two on Chromosomes 14 and 21 showed a positive effect (increased weight) (Figure 4). For BW, seven significant regions were identified across six chromosomes. Four regions, located on Chromosomes 2, 6, and 28, showed a negative effect, whereas one region on Chromosome 11 showed a positive effect. Additionally, three other regions with positive effects were found on Chromosomes 3 and 13, as well as another also identified on Chromosome 11 (Figure 5).

### 3.4 Annotated Genes

A total of 269 genes were identified within the significant genomic regions. Of these, 41 genes were associated with the analysed traits based on functional annotation (Table 7).

**TABLE 7:** Annotated genes located ±0.5 Mb around SNPs with –log_10_(*p*) > 3 and their direction of effect (+ for favourable and – for unfavourable) on significant traits (AFC, BW, W210).

Trait	BTA	Start (in Bps)	End (in Bps)	Genes	Effect over trait
AFC	1	137,170,300	137,741,631	*NPHP3, ACKR4, ACP3, CPNE4, NUDT16, NEK11*	+
	2	3,435,871	6,700,805	*PROC, CYP27C1, NAB1, MSTN, ORMDL1, COL5A2*	−
	4	67,489,709	68,253,462	*TRIL*	−
	5	94,753,942	101,124,171	*RERG, MGP, CDKN1B, CREBL2, YBX3, KLRK1, CLEC7A, CLEC12B, GDF3, NANOG, C3AR1*	+
	17	38,207,944	41,135,724	*FAT4, FGF2, IL21, IL2, TRPC3*	+
BW	2	6,216,138	8,305,740	*MSTN, COL5A2, COL3A1*	−
	3	60,954,055	61,920,582		+
	6	38,415,398	39,438,580	*SLIT2*	−
	11	86,711,869	88,537,653	*GREB1, ROCK2, ITGB1BP1, ID2*	+
		20,202,555	20,558,025	*CYP1B1*	−
	13	33,903,159	34,808,642	*ZEB1*	+
	28	36,504,079	36,733,035	*NRG3*	−
210W	2	6,068,315	7,046,478	*MSTN, COL5A2, COL3A1*	−
	14	78,990,321	80,016,715		+
	21	58,188,882	60,115,792	*ASB2, TCL1A*	+

Abbreviations: AFC, age at first calving; BW, birth weight; CI, calving interval; W210, weight at 210 days.

Regions associated with increases in AFC and decreases in BW and W210 were identified. We detected the most relevant region in chromosome (BTA) 2 (3,435,871–6,700,805), including *NAB1* (NGFI‐A Binding Protein 1), which is located near *MSTN* (Myostatin), within the same genomic region, and *COL5A2* (Collagen Type V Alpha 2 Chain). This region showed a difference of −13.46 kg (±1.60 kg) in W210 and − 0.50 kg (±0.04 kg) in BW, and furthermore, it showed a 13.46‐day (±1.60‐day) delay in AFC. A second notable region located on BTA6 (38415398–39438580) included *SLIT2* (Slit Guidance Ligand 2) and showed a difference of −0.530 ± 0.018 kg in BW. Other regions, such as that on BTA1 (137170300–137741631), including *CPNE4* (Copine 4), on BTA5 (94753942–101124171), including *RERG* (RAS‐Like Estrogen‐Regulated Growth Inhibitor), *YBX3* (Y‐Box Binding Protein 3, also known as csda1) and *NANOG* (Nanog Homeobox), and on BTA17 (38207944–41135724), including *FGF2* (Fibroblast Growth Factor 2), showed a negative effect on AFC, increasing the number of days at first calving.

Other genomic regions were associated with increases in BW, BTA13 (33903159–34808642) included *ZEB1* (Zinc Finger E‐Box Binding Homeobox 1), and in W210, BTA21 (58188882–60115792) included two immune‐related genes: *ASB2* (Ankyrin Repeat and SOCS Box Containing 2) and *TCL1A* (TCL1 Family AKT Coactivator A). On the other hand, a region decreasing AFC, in addition to that outlined before on BTA2, mapped on BTA4 (67489709–68253462) showing a 23.93‐day advance and including *TRIL* (TLR4 Interactor with Leucine Rich Repeats).

The most significantly enriched terms for each region and trait are listed in Table 8, along with their adjusted *p* values and the corresponding annotation database (GO:CC, GO:MF, GO:BP, REAC, MIRNA, HP, KEGG). In general, enriched terms were related to transcriptional regulation, extracellular matrix organisation, immune response and metabolic processes. Region 2:3,435,871–6,700,805 was enriched in terms related to AFC. The miR‐27 family of microRNAs, including ‘bta‐miR‐27b’ located in this region, has been reported in tissues related to reproductive function and early pregnancy in cattle. Additionally, region 4:67,489,709–68,253,462 showed enrichment for the term ‘lipopolysaccharide receptor complex’. Region 5:94,753,942–101,124,171 was annotated with terms related to taste perception activity. For BW, enriched terms were identified in three regions. Region 2:6,216,138–8,305,740 showed enrichment related to collagen structure and connective tissue. Region 11:86,711,869–88,537,653 was enriched in terms related to regulation of the ‘G1/S transition of the mitotic cell cycle’, whereas region 11:20,202,555–20,558,02 showed enrichment in the ‘estrogen receptor pathway’. For the 210 W trait, two regions with significant enrichment were found: 14:78,990,321–80,016,715, enriched in ‘nitrogen metabolism’ and related terms, and 21:58,188,882–60,115,792, enriched in terms related to peptidase activity.

**TABLE 8:** Functionally enriched terms identified in genomic regions significantly associated with each trait.

Trait	Genomic region (BTA:start‐end)	Enriched term	Adjusted *p*	Database
AFC	2:3,435,871–6,700,805	RNA Polymerase II Transcription Elongation	5.744 × 10^−3^	REAC
		RNA Polymerase II Pre‐Transcription Events	1.767 × 10^−2^	REAC
		bta‐mir‐27b	4.997 × 10^−2^	MIRNA
	4:67,489,709–68,253,462	Lipopolysaccharide receptor complex	4.980 × 10^−2^	GO:CC
	5:94,753,942–101,124,171	Bitter taste receptor activity	4.776 × 10^−15^	GO:MF
		Taste receptor activity	9.257 × 10^−14^	GO:MF
		Detection of chemical stimulus involved in sensory perception of bitter taste	1.631 × 10^−13^	GO:BP
		Detection of chemical stimulus involved in sensory perception of taste	8.610 × 10^−13^	GO:BP
		Sensory perception of bitter taste	1.761 × 10^−12^	GO:BP
		Taste transduction	1.545 × 10^−11^	KEGG
BW	2:6,216,138–8,305,740	Fibrillar collagen trimer	8.254 × 10^−4^	GO:CC
		Banded collagen fibril	8.254 × 10^−4^	GO:CC
		Complex of collagen trimers	2.491 × 10^−3^	GO:CC
		Osteoarthritis	5.610 × 10^−3^	HP
		SMAD binding	3.237 × 10^−2^	GO:MF
	11:86,711,869–88,537,653	Regulation of G1/S transition of mitotic cell cycle	1.555 × 10^−4^	GO:BP
		Regulation of cell cycle G1/S phase transition	2.859 × 10^−4^	GO:BP
		G1/S transition of mitotic cell cycle	5.222 × 10^−4^	GO:BP
	11:20,202,555–20,558,025	Oestrogen receptor pathway	4.984 × 10^−2^	WP
210W	14:78,990,321–80,016,715	Nitrogen metabolism	5.059 × 10^−12^	KEGG
		Reversible hydration of carbon dioxide	2.453 × 10^−10^	REAC
		Carbonate dehydratase activity	2.949 × 10^−7^	GO:MF
		Hydro‐lyase activity	2.719 × 10^−5^	GO:MF
	21:58,188,882–60,115,792	Serine‐type endopeptidase inhibitor activity	7.554 × 10^−4^	GO:MF
		Endopeptidase inhibitor activity	2.887 × 10^−3^	GO:MF
		Peptidase inhibitor activity	3.705 × 10^−3^	GO:MF

Abbreviations: AFC, age at first calving; BW, birth weight; CI, calving interval; W210, weight at 210 days.

## 4 Discussion

Because of the lack of methodological standardisation, the comparison of ROH measures across studies should be evaluated cautiously, even though it can offer a helpful context. The number, length and distribution of ROH detected are greatly influenced by variations in software tools (PLINK, detectRUNS), detection techniques (sliding window or consecutive runs), ROH parameter settings (minimum SNPs/ROH, maximum gap length, allowed heterozygosity and missing data), but also genotyping density and chip platform. Therefore, these comparisons are not meant to be direct quantitative contrasts, even though they are included here to offer an overview of reference. In order to acknowledge the diversity of ROH research and the need for additional standardisation in the field, they instead seek to contextualise the findings within the large body of literature.

The average number of ROHs per animal in the RG population (79.03) is notably lower than that reported in other studies, such as Xu et al. (2019), which found 153 ROHs per animal in local Chinese cattle or Zhang, Guldbrandtsen, et al. (2015), which reported 715 ROHs per animal in Danish Dairy Cattle. However, the average size of ROHs in the RG breed (2.53 Mb) is larger than in Chinese breeds (1.22 Mb) and Danish breeds (0.75 Mb). For another Spanish native breed, the Asturiana de los Valles (AV), the average was 100.31 ROHs per animal, with an average length of 6.7 Mb, using the same minimum ROH length parameter of 1 Mb (Cortes et al. 2024). The number of ROHs per individual provides insights on the inbreeding levels within a breed. For instance, the Chinese and Danish breeds exhibit a higher number of ROHs per animal, which correlates with higher *F*_ROH_ values—ranging from 0.031 to 0.115 for the former and averaging 0.195 for the latter—compared to the RG and the AV breed (0.084 and 0.076, respectively).

The estimates of variance components and heritabilities are consistent with previous findings for this population (Martinez‐Castillero et al. 2021) as well as other beef cattle populations (Brzáková et al. 2020; Ríos‐Utrera and Van Vleck 2004). The Pearson and Spearman correlations between *F*_ROH_ and *F*_PED_ were 0.66 and 0.52, respectively. These estimates are comparable to those observed in the AV (Cortes et al. 2024), Alpine–Gray (Gomez Proto et al. 2024) and Holstein, Jersey and Red Danish Cattle (Zhang et al. 2015). Relatively low correlations were expected, given the higher accuracy of genomic evaluation for measuring inbreeding and the deficiencies in pedigree quality within the RG breed. The correlation between *F*_GRM_ and *F*_PED_, according to Spearman's method, was 0.00, whereas using Pearson's method it was 0.44. This difference can be mainly attributed to the distinct calculations underlying each measure. Although Spearman assesses rank correlation (i.e., individuals with high *F*_GRM_ should also have high *F*_PED_), Pearson reflects an overall linear relationship (i.e., average inbreeding may increase with *F*_PED_). This indicates that the relationship between these two coefficients is not monotonic, and *F*_PED_ may overestimate or underestimate inbreeding due, for example, to poor pedigree quality.

Despite the substantial number of records analysed, the population achieves only 3.79 ECG and 1.64 FCG. This ECG is higher than that reported by Cañas‐Álvarez et al. (2014) for RG (3.08), but it remains low in comparison with the other indigenous cattle breeds in the study, being below Avileña (3.99) and Pirenaica (4.62). It is also lower than most other native Galician breeds (‘Morenas do Noroeste’), such as Cachena (4.2), Caldelá (4.1) and Frieiresa (3.8) (García‐Atance et al. 2023), despite RG showing a larger census, a higher degree of professionalisation, and broader distribution. The correlations between *F*_PED_ and *F*_ROH_ tend to decrease as the size threshold of the ROH (2, 4, 8 and 16 Mb) used to calculate the coefficient increases. This phenomenon may be attributed to the fact that a large percentage of individuals have *F*_ROH_s of zero as the length increases.

It is also noteworthy that a low correlation was observed between the two molecular inbreeding estimators, *F*_ROH_ and *F*_GRM_. *F*_GRM_ depends on initial allele frequencies giving greater weight to rare alleles, which means that rare homozygotes contribute more to inbreeding than common homozygotes. In contrast, *F*_ROH_ does not rely on allele frequencies (McQuillan et al. 2008). This poor correlation, as reported elsewhere (Marras et al. 2015; Mastrangelo et al. 2016; Mulim et al. 2022; Zhang, Calus, et al. 2015), can also be attributed to the properties of the **G** matrix, which is based on individual loci rather than chromosomal segments, as is the case with *F*_ROH_ (Zavarez et al. 2015).

The strength of the relationship between different inbreeding estimators in the literature has traditionally been assessed using Pearson correlation (Cortes et al. 2024; Rodríguez‐Ramilo et al. 2015; Xu et al. 2019; Zhang, Calus, et al. 2015; Zhang, Guldbrandtsen, et al. 2015). However, as *F* (*F*_ROH_, *F*_PED_ or *F*_GRM_) values are not normally distributed, Pearson correlation may overestimate the true association (Gurgul et al. 2016). Therefore, the nonparametric Spearman correlation was chosen as an alternative. It is also important to recognise that the use of different correlation coefficients, combined with the variation of *F*_ROH_ estimations—arising from differences in the number of genotyped animals and marker density even within the same breed (Carrara et al. 2024)—along with inconsistencies in defining ROH (Peripolli et al. 2017), complicate the comparison of studies.

Accordingly, it is clear that pedigree quality will influence the estimation of inbreeding depression (Cassell et al. 2003). Using ROHs for this purpose can provide a more effective, consistent and straightforward understanding of inbreeding depression. However, it is important to consider the potential challenges associated with this method of predicting inbreeding. On the one hand, the traits under study are influenced by artificial selection, which means that in some cases, homozygosity—and, consequently, the presence of ROHs—can be beneficial for production (Bjelland et al. 2013). On the other hand, although genotyping errors may occur, they are less likely with current technologies, and in this study, it is very unlikely that such errors exceed those associated with pedigree data. Nevertheless, these errors may lead to an underestimate of *F*_ROH_, as incorrectly genotyped SNPs classified as heterozygous can disrupt longer ROHs or generate shorter ones. To mitigate this potential issue, this study followed the recommendations of Ferenčaković et al. (2013) regarding the allowance of heterozygotes.

The effects of inbreeding depression have been studied through ROH in numerous species and across various traits, primarily impacting reproductive performance (Laseca et al. 2024). Bjelland et al. (2013) reported that a 1% increase in F_ROH_ in Holstein cattle is associated with an increase of 1.72 days open and a 0.82% decrease in conception rate. Similarly, Martikainen et al. (2018) noted a one‐day increase between the first and last insemination with a 10% increase in *F*_ROH_ in Finnish cattle. Cassell et al. (2003), also studying Holstein, have found a negative but non‐significant effect of inbreeding on reproductive performance. This finding is supported by Pryce et al. (2014) and Cortes et al. (2024). In our study, we were able to detect significant inbreeding depression for the reproductive traits (AFC and CI), and the estimates show an undesirable increasing trend as the size of the ROHs increases, indicating more recent inbreeding, this effect being statistically significant in all cases (> 0.01 for AFC and CI). The strongest significance was observed with more recent inbreeding, with *p* < 0.005 for *F*_ROH>8_ in AFC and *F*_ROH>16_ in CI. For productive traits (BW and W210), we could not find significant effects for RG, although the trends were consistently negative, as previously reported by Bjelland et al. (2013) and Cortes et al. (2024) for birthweight.

The increasing significance observed with larger ROH sizes is expected and has been reported by other authors. Cortes et al. (2024) showed a significant effect of inbreeding depression on preweaning average daily gain and weaning weight adjusted at 180 days, but only for *F*_ROH_ calculated from ROHs greater than 17 Mb, with no significance observed for ancient (*F*_ROH<4_) or medium (*F*_ROH(4,17]_) inbreeding. Similarly, Pryce et al. (2014) reported that shorter ROHs (< 60 SNPs or 3.5 Mb) were not associated with milk production in Holstein and Jersey cattle, whereas larger ROHs exhibited a deleterious effect, indicating a stronger unfavourable effect.

As mentioned earlier, the effects of homozygosity in traits under selection can be undesirable due to inbreeding depression or beneficial as a result of artificial selection (Bjelland et al. 2013). This trade‐off can influence the estimation of inbreeding depression with *F*_ROH_. To gain a deeper understanding of the architecture of inbreeding depression, we analysed the effects associated with the presence or absence of each SNP within ROHs, and we have identified genomic regions associated with unfavourable and beneficial effects.

### 4.1 Annotated Genes Associated With Target Traits

The most interesting region on BTA2 included the *MSTN* (Myostatin) gene involved in the double‐muscle phenotype and responsible for significant muscle development (Grobet et al. 1997), and *COL5A2*, involved in skeletal development, myogenesis and muscle growth (Shen et al. 2021). This region showed a negative effect on BW and W210, which could be explained by selection on AFC, as a ROH island was identified overlapping this region in RG breed, potentially leading to an unfavourable haplotype in the *MSTN* gene for weight traits (Hervás‐Rivero et al. 2023). Also, a region on BTA6 negatively associated with growth included *SLIT2*, a gene that has been associated with various weight traits, including internal organ weight in Simmental cattle (An et al. 2018), bone weight in beef cattle (Niu et al. 2021), and birth, yearling and weaning weights in US Red Angus cattle (Smith et al. 2022), as well as birth weight in US Gelbvieh cattle (Smith et al. 2019). Several other genes mapping on genomic regions negatively associated with target traits, such as *CPNE4* on BTA1, a gene influencing growth, size, muscle, and bone development across various farm animal species, including cattle (Barbato et al. 2020; Gouveia et al. 2014; Jahuey‐Martínez et al. 2016); *RERG*, a gene implicated in steroid production and muscle growth (Neves et al. 2019), *YBX3* is involved in muscle development and protein metabolism (Saito et al. 2011), and *NANOG* is associated with embryonic development and cellular response to growth factors, with implications for fertility in tropical bulls (Porto‐Neto et al. 2023), all three of them on the BTA5 region. Finally, *FGF2*, a gene linked to fertility traits in cattle (Oikonomou et al. 2011), is mapped on BTA17.

Among the genes with favourable effects on target traits, *ZEB1* mapping on BTA13, plays a significant role in the regulation of reproduction (Liu et al. 2017); *ASB2* on BTA21, proved to be a negative regulator of muscle growth in salmon (Bower and Johnston 2010), although *TCL1A* has been involved in immunity through T‐ and B‐cell development (Kang et al. 2005). Both genes have been associated with weight traits in cattle (Iung, et al. 2018; Mudadu et al. 2016). Selection of haplotypes involving a favourable combination of allelic variants of these genes, improving the immune system, could underlie this association. Finally, *TRIL* mapping on BTA4 can lead to increased feed intake when inhibited, due to its role in leptin sensitivity, a hormone regulating feed behaviour (Moura‐Assis et al. 2021; Nkrumah et al. 2004).

### 4.2 Enrichment Analysis

Regarding functional enrichment, for AFC many relevant terms were found. The miR‐27 family of microRNAs has been associated with, and detected in, various tissues related to reproductive function (Salilew‐wondim et al. 2014) and early pregnancy (Ioannidis and Donadeu 2016) in cattle. Thus, ‘bta‐miR‐27b’ holds potential for correlation with fertility traits (MacLeay et al. 2025), notably with AFC. Additionally, as RNA polymerase II transcribes all protein‐coding genes, its activity is essential for numerous biological processes, including those that regulate early developmental stages and embryonic viability (Kovalská et al. 2010). These processes, in turn, have a direct impact on fertility (Reese et al. 2020). The enriched term ‘lipopolysaccharide receptor complex’ suggests a potential role of innate immune function and uterine/ovarian inflammatory responses in determining reproductive maturation (Bromfield and Sheldon 2013; Dickson, Sheldon, & Bromfield, Dickson et al. 2022). Finally a correlation with ‘taste perception activity’ (bitter taste receptor, detection of chemical stimulus involved in bitter taste, taste transduction, etc.) was found. Although palatability can influence feed intake, and intake may in turn be associated with reproductive capacity (Damiran et al. 2018; Randel and Welsh 2013), these relationships are indirect, vague and would require further investigation to be properly established.

Regarding BW, the relation with collagen structure and connective tissue in general stood out. This is supported by enriched terms indirectly related, such as ‘osteoarthritis’, suggesting the involvement of pathways associated with tissue remodelling, but others directly associated, such as ‘SMAD binding’ with a role in collagen expression (Ellis et al. 2003). As a structural protein, collagen is closely associated with the maintenance of placental growth and its functionality (Breeveld‐Dwarkasing et al. 2003; Wawrzykowski et al. 2025). Also enriched terms related to the regulation of the G1/S transition of the mitotic cell cycle appeared. Although this process could be associated with cellular proliferation or tissue development, the terms identified are very general and linked to fundamental biological mechanisms. The ‘estrogen receptor pathway’ has been associated with placental development and growth due to its presence in bovine placentomes (Schuler et al. 2002), suggesting a possible link with foetal development. Additionally, Meyer et al. (2007) suggest that the oestrogen receptor alpha is key for cellular growth, which could be indirectly related to BW. In animal science, a major breakthrough was made by Rothschild et al. in (1996), when they identified a specific gene (the oestrogen receptor locus) with a significant effect on litter size in pigs.

Although direct evidence linking ‘nitrogen metabolism’, ‘reversible hydration of carbon dioxide’, ‘carbonate dehydratase activity’ and ‘hydro‐lyase activity’ with weight at 210 days in bovines is scarce, efficient nitrogen metabolism is a well‐established determinant of growth rate and body composition in cattle (Zanton and Heinrichs 2008), which likely contributes to variation in post‐natal growth up to 210 days. Finally, the superfamily of serpins—which includes ‘serine‐type endopeptidase inhibitor activity’—has been previously associated with various growth‐related traits in cattle (Yang et al. 2020). These inhibitors may modulate proteolysis and tissue remodelling, thereby influencing growth dynamics during postnatal development.

The relationships described above between genes and enriched terms with traits of interest in the RG breed represent a preliminary approach, and accordingly these results should be interpreted with caution. Further research is necessary to strengthen and validate these potential correlations.

## 5 Conclusions

The present study provides an analysis of runs of homozygosity and their impact on inbreeding depression in the Rubia Galega cattle. A predominance of ancient inbreeding was found, through the number and length of ROHs, and the inbreeding coefficient based on ROHs, *F*_ROH_, appeared as a moderately high and more informative metric than pedigree‐based estimates, especially given the limited pedigree depth of the population. Significant inbreeding depression was found in reproductive traits, age at first calving (AFC) and calving interval (CI), with more recent inbreeding showing the strongest negative effects. Genomic regions associated with both favourable and unfavourable effects over fertility and growth were identified. Genes such as *MSTN*, *COL5A2*, *SLIT2* and *FGF2* appear in regions linked to detrimental effects on growth and reproductive traits, whereas others such as *ZEB1*, *ASB2* and *TCL1A* were associated with improved performance. Biological processes related to tissue development, immune function or reproductive regulation were found in the functional and enrichment analyses, supporting the relevance of the significant genetic regions. The importance of ROH‐based analysis in characterising inbreeding and its effects is demonstrated by all these findings. Furthermore, the discovery of certain genetic areas linked to inbreeding depression offers information that may help guide targeted selection strategies. To reduce the detrimental impacts of inbreeding and improve performance in the RG breed, further research should validate these locations and evaluate their possible application in breeding programs.

## Funding

Funding was provided by Xunta de Galicia local government (Spain) grant for the support of pilot projects for the development of new products, practices, processes and technologies in the agroforestry field (MR331A) and support for Excellence Research Groups (ED431C 2022/33). Nicolás Mejuto‐Vazquez carries out their research activities thanks to the collaboration agreement between USC and the Fundación Caixa Rural Galega—Tomás Notario Vacas—for the funding of a predoctoral contract. David López‐Carbonell is financed by a DGA (Diputacion General de Aragón) predoctoral grant.

## Conflicts of Interest

The authors declare no conflicts of interest.

## Data Availability

The data that support the findings of this study are available from the corresponding author upon reasonable request.
